# The Administration of Heat Shock Protein-70 Bacterial Homolog (DnaK) Improves the Cumulative Survival and the Expression of Immune-Related Genes in Gnotobiotic Full-Sibling Sea Bass Larvae Challenged with *Vibrio anguillarum*

**DOI:** 10.3390/ani15111655

**Published:** 2025-06-04

**Authors:** Eva Vallejos-Vidal, Camino Fierro-Castro, María Jesús Santillán-Araneda, Merari Goldstein, Sebastián Reyes-Cerpa, Joan Carles Balasch, Ali Reza Khansari, Kristof Dierckens, Peter Bossier, Lluis Tort, Felipe E. Reyes-López

**Affiliations:** 1 Fish Health and Integrative Physiogenomics Research Team, Centro de Biotecnología Acuícola, Facultad de Química y Biología, Universidad de Santiago de Chile, Santiago 9170002, Chile; eva.vallejosv@usach.cl (E.V.-V.); maria.santillan@usach.cl (M.J.S.-A.); merari.goldstein@usach.cl (M.G.); 2 Núcleo de Investigación en Producción y Salud de Especies Acuáticas (NIP-SEA), Facultad de Medicina Veterinaria y Agronomía, Universidad de Las Américas, La Florida, Santiago 8250122, Chile; 3 Centro de Nanociencia y Nanotecnología CEDENNA, Universidad de Santiago de Chile, Santiago 9170002, Chile; 4 Departamento de Biología Molecular, Área de Genética, Facultad de Ciencias Biológicas y Ambientales, Universidad de León, 24071 León, Spain; c.fierro@unileon.es; 5 Centro de Genómica y Bioinformática, Facultad de Ciencias, Ingeniería y Tecnología, Universidad Mayor, Santiago 8580745, Chile; sebastian.reyes@umayor.cl; 6 Escuela de Biotecnología, Facultad de Ciencias, Ingeniería y Tecnología, Universidad Mayor, Santiago 8580745, Chile; 7 Department of Cell Biology, Physiology and Immunology, Faculty of Biosciences, Universitat Autònoma de Barcelona, 08193 Bellaterra, Spain; joancarles.balasch@uab.cat (J.C.B.); lluis.tort@uab.cat (L.T.); 8 Department of Biological & Environmental Sciences, University of Gothenburg, 41390 Göteborg, Sweden; ali.khansari@bioenv.gu.se; 9 Laboratory of Aquaculture & Artemia Reference Center (ARC), Ghent University, Rozier 44, B-9000 Gent, Belgium; kristof.dierckens@ugent.be (K.D.); peter.bossier@ugent.be (P.B.)

**Keywords:** aquaculture, HSP70, DnaK, immune response, sea bass larvae, *Vibrio anguillarum*

## Abstract

Heat shock proteins (HSPs), particularly HSP70, play a vital role in fish immune defense against pathogens. The administration of DnaK (bacterial homolog of HSP70) may be a strategy to potentiate the immune response and survival of aquatic organisms. This study evaluates the effect of cells overexpressing DnaK on mortality and immune-related gene expression in gnotobiotic sea bass larvae challenged with *Vibrio anguillarum*. Larvae were subjected to different treatments: NB (no bacteria), YS0 (*E. coli* with no plasmid), YS1 (*E. coli* expressing truncated DnaK), and YS2 (*E. coli* expressing DnaK), and then infected with *V. anguillarum* at 7 days post-hatching (dph). Mortality was monitored, and RT-qPCR was used to evaluate immune gene expression at 0, 18, 24, 36, and 120 hpc. While no significant variations were recorded in the non-challenged larvae, constant and sustained mortality was observed in challenged larvae from 60 to 120 hpc. However, lower mortality was observed in the larvae treated with DnaK. DnaK treatment promoted the expression of antimicrobial (*hepcidin*, *transferrin*) and chemotaxis genes (*ccl4*), which was further enhanced after a challenge with *V. anguillarum*, in conjunction with the modulation of *il1β* and *il-8* at 120 hpc. These findings suggest that DnaK induces a potent innate immune response, improving survival against *V. anguillarum* and supporting its potential use as a disease-preventive strategy in aquaculture.

## Simple Summary

Heat shock proteins (HSPs) are a family of highly conserved proteins identified in diverse living organisms that protect cells from stress. HSPs also participate in the activation of defense mechanisms in the body, promoting the activation of the immune system. Previous studies have shown that the administration of DnaK (bacterial homolog of HSP70) may be a strategy to potentiate the immune response and survival of aquatic organisms against bacterial infections. Thus, this study aimed to evaluate the effect of cells overexpressing DnaK on the mortality and immune-related gene expression in gnotobiotic sea bass larvae challenged with *Vibrio anguillarum*. The results showed lower mortality in the sea bass larvae treated with DnaK and challenged with *V. anguillarum*. This response would be promoted by an increase in the expression of genes associated with antimicrobial, pro-inflammatory, and chemotaxis responses. These findings suggest that DnaK administration induces a potent innate immune response, enhancing the survival of sea bass larvae challenged with *V. anguillarum*. The use of DnaK could be used as a pathogen resistance agent, allowing the prevention of diseases in aquatic organisms used for aquaculture.

## 1. Introduction

Stress is a state in which intrinsic and/or extrinsic stressors disrupt or threaten the organism’s homeostasis, which may harm its integrity [1,2]. Once the stressor is perceived, the hypothalamic-pituitary-interrenal (HPI) axis is activated, releasing cortisol, considered the main glucocorticoid [3]. The physiological stress response mediates the release of stress hormones and is accompanied by a rapid increase in the synthesis of stress-specific proteins to regulate the overall reaction to this allostatic load. Heat shock proteins (HSPs) synthesis is also promoted at the cellular level [4,5,6]. HSPs are a family of highly conserved molecular chaperones identified in diverse living organisms, from bacteria to humans [7]. They are essential in various biological processes, such as the transporting, folding, and assembly of degraded or misfolded proteins. Moreover, several reports have shown that HSPs participate in host defense by potentiating inflammatory responses and promoting cytokine production [8,9,10,11,12]. Due to these immunomodulatory properties, HSPs have been explored as potential antigens for infection control [13].

The members of this superfamily are classified according to their relative molecular mass, structural homology, and function [9]. Three HSP families are described: HSP90 (85–90 kDa), HSP70 (68–73 kDa), and low molecular weight HSP (16–47 kDa) [14]. The HSP70 family is the most abundant and best studied [10]. HSP70 is involved in numerous functions in various cellular processes, including protecting proteins against stress, and has been identified in several fish species [7,9]. Traditionally, this protein is considered a biomarker of environmental stress in teleost fish. However, its expression has been documented to increase with dietary additives in aquaculture [15,16]. According to previous studies, the use of dietary probiotics and prebiotics leads to increased expression of HSP70 in the intestine of rainbow trout [17] and Asian seabass (*Lates calcarifer*) [18], respectively. This upregulation has improved fish health and enhanced resistance to pathogen infections. In fact, HSP70 has gained increasing attention in aquaculture fish species for its involvement in immune response against pathogens [19], enhancing resistance against *Vibrio campbellii* and *V. proteolyticus* [1].

The prokaryotic HSP70, also known as DnaK, can induce innate immune responses and survival in several aquatic organisms, including fish, shrimp, and mollusks, against different bacterial infections [20,21]. DnaK homologs are present in most bacterial species and play protective and regulatory roles in the immune response [22]. Such is the case of DnaK from *Mycobacterium tuberculosis*, which acts as an immunomodulator during infection by polarizing macrophages to an M2 phenotype [22]. These microbial proteins have been widely used as relevant antigens in defense against infectious diseases by potent immune system activators [23]. The existing literature on the in vivo effect of DnaK in fish has been mainly focused on administering the recombinant or encapsulated protein through microparticles [20,24]. However, it is essential to note that microparticle production and application entail significant costs and involve more complex logistics compared to other administration techniques.

Fish represent excellent models for studying HSP expression, as various stressors are encountered in their environment and/or can be exposed to them in an experimental setting [25]. In response to stress, teleosts were the first vertebrates to develop a response involving a complex network of signals from the central regulatory system: neuronal, endocrine, and immune, mainly through the HPI and Brain-Sympathetic-Chromaffin (BSC) axes and their interaction with networks associated with the defense response [2,26]. Thus, the perception of a stressor promotes the activation of multiple mechanisms in the central nervous system that trigger the secretion of specific neuropeptides and hormones responsible for the immune effector action [27]. Additionally, several studies have demonstrated that pro-inflammatory cytokines (IL-6, IL-1, TNFα) play an active role in regulating certain stress hormones, demonstrating the interconnectedness of these regulatory systems and their role in fish physiology [2,28].

During the fish life cycle, the larval stage undergoes differentiation and activation of many organs. This process requires early neuro-immune-endocrine integration using signaling molecules that may be different from those of adult fish. The immune-endocrine system has been studied in several fish species, including the sea bass (*Dicentrarchus labrax*) [20]. Sea bass is a species of great commercial interest; being one of the three main species bred in Europe, it is also among the most studied models of teleost fish [14]. To achieve high production rates, these fish may be subjected to stressful conditions that trigger the appearance of pathogenic diseases such as vibriosis, mainly caused by *Vibrio anguillarum* in these fish [29].

Infectious diseases are one of the most critical issues for the aquaculture industry, resulting in significant economic losses due to high mortality. *V. anguillarum* (the etiological agent of vibriosis) is a gram-negative marine bacterium considered pathogenic for several fish species relevant to the aquaculture industry, including sea bass [30]. To date, few treatments are available to improve the immunity and health of fish infected with *V. anguillarum*. For this reason, this study aimed to evaluate the potential protective action of cells overexpressing DnaK and its effect on the cumulative mortality and immune-related gene expression pattern in gnotobiotic full-sibling sea bass larvae challenged with *V. anguillarum*. The study was conducted under gnotobiotic conditions because this approach enhanced the experimental reproducibility [20]. Experimentally, sea bass larvae were incubated on day 3 post-hatching with a unique pulse of one of the following treatments: YS0 (*E. coli* without plasmid), YS1 (*E. coli* expressing truncated DnaK), and YS2 (*E. coli* expressing DnaK). Then, on day 7 post-hatching, sea bass larvae were bath-incubated with *V. anguillarum* strain HI610. The cumulative survival and the expression of a set of immune-related genes were evaluated at different time points (0, 18, 24, 24, 36, and 120 h post-challenge). This study sheds light on the mechanisms modulated by DnaK in sea bass larvae exposed to a pathogen relevant to the aquaculture industry.

## 2. Materials and Methods

### 2.1. Bacterial Strain and Culture Conditions

*Vibrio anguillarum* strain HI 610 serovar O2a was used to evaluate the effect of DnaK as an immune protective molecule. The bacteria were initially isolated by O. Bergh (Institute of Marine Research, Bergen, Norway) from cod with vibriosis symptoms at the Parisvatnet research facility in Norway. The strain was made rifampicin-resistant by natural selection, allowing its viability in the axenic system. The bacteria were grown in 10% marine broth (Difco Laboratories, Detroit, MI, USA) with the addition of NaCl to obtain the same salinity as the water in the fish larvae experiment (36 g L^−1^) and kept on a horizontal shaker at 150 rpm at 16 °C. The density of the bacterial suspension was determined with a spectrophotometer (Genesys 20, Thermospectronic, Thermo Electron, Waltham, MA, USA) at 550 nm according to the McFarland standard (BioMérieux, Marcy L’Etoile, France).

### 2.2. Induction and Synthesis of DnaK

The plasmid constructs for synthesizing DnaK have been developed and described elsewhere, as has their protective effect against bacterial infection [31]. Briefly, the DnaK cDNA sequence from *E. coli* str K-12 substr. MG1655 (GenBank: AIZ92815.1) was amplified with specific primers, cloned into the TOPO cloning vector, and transformed into *E. coli* One Shot TOP10 cells and grown on LB agar containing 100 μg·mL^−1^ ampicillin at 37 °C. A bacterial-positive clone containing DnaK cDNA (henceforth YS2) was isolated from the LB plates. A construct containing a short DNA fragment with two in-frame stop codons that make protein production unviable was used as a negative control for DnaK expression (henceforth YS1). In addition, *E. coli* with no plasmid (henceforth YS0) was also included in the study. All bacterial cell cultures were grown in LB broth at 37 °C. Overnight cultures were diluted (1:50) in 5 mL fresh LB broth and incubated at 37 °C (220 rpm) until the cultures reached an OD_600_ value of 0.5–0.7. Then, DnaK synthesis was induced with 0.5 mg·mL^−1^ ʟ-arabinose for 4 h. The presence of DnaK in the culture was verified by analyzing the protein extract by SDS-PAGE and Western blot. After induction, bacteria were harvested by centrifugation at 2200× *g* for 15 min at room temperature. Pellets were washed once with seawater (previously autoclaved and filtered) and then suspended in 30 mL of autoclaved seawater. The bacterial cell culture was quantified spectrophotometrically at 550 nm and used to immediately feed the sea bass larvae at 4 days post-hatching (dph) using a concentration of 10^7^ cells/mL, as described in previous studies [31,32].

### 2.3. Protein Extraction, SDS-PAGE, and Western Blot to Verify DnaK Synthesis

The bacterial suspension was homogenized with 0.1 mm diameter glass beads in cold buffer K (150 mm sorbitol, 70 mm potassium gluconate, 5 mm MgCl_2_, 5 mm NaH_2_PO_4_, 40 mm HEPES, pH 7.4) containing protease inhibitor cocktail (Sigma-Aldrich, Diegem, Belgium) at the highest recommended level. Samples were centrifuged at 2200× *g* for 1 min at 4 °C. The supernatant containing the crude protein extract was then collected to determine its concentration using the Quick Start Bradford Protein Assay (Bio-Rad, Hercules, CA, USA). DnaK was also purified as described elsewhere [20] and used as a positive control. For SDS-PAGE, 50 µg of total protein extract was combined with 2 × SDS polyacrylamide gel buffer (ratio 1:1), incubated at 95 °C for 5 min, and loaded in 10% SDS polyacrylamide gels (Bio-Rad) and then stained with Coomassie Biosafe (BioRad). The proteins were transferred to polyvinylidene fluoride membranes (PVDF, Bio-Rad) to detect DnaK synthesis by Western blot. Membranes were incubated for 60 min with blocking buffer (PBS 1×, 0.2% *v*/*v* Tween-20, 5% *w*/*v* BSA). DnaK detection was performed with the monoclonal antibody 8E2/2 mouse anti-DnaK at a dilution of 1:1000 (Stressgen Bioreagents, Victoria, BC, Canada), and the horseradish peroxidase-conjugated donkey anti-mouse IgG was used as a secondary antibody at a dilution of 1:4000 (Affinity Bioreagents, Golden, CO, USA). Detection was performed with a chemiluminescent western blot detection kit (Bio-Rad). Images were captured with the ChemiDoc MP system (Bio-Rad).

### 2.4. Gnotobiotic Full-Sibling Sea Bass Larvae

Sea bass eggs of 2 days post-fertilization were obtained from Ecloserie Marine in Gravelines, France. One batch of full-sibling eggs was acquired for this experiment. Upon arrival, the eggs were acclimatized in UV-sterilized seawater for 4 h in a cylindro-conical tank at 16 ± 1 °C and a salinity of 36 g L^−1^. The disinfection of full-sibling sea bass eggs, hatching, and testing for axenity were performed according to Dierckens et al. (2009) [33]. Handling and procedures, including hatching, stocking, and treatments, were carried out under a laminar flow hood and in a temperature-regulated room (16 ± 1 °C) with dim light (100 lux), as described elsewhere [20].

### 2.5. Full-Sibling Sea Bass Larvae Incubated with E. coli Overexpressing DnaK

On day 3 post-hatching (3 dph), n = 12 full-sib sea bass larvae were stocked individually in each 10 mL sterile screw cap vial (n = 4 replicate vials per treatment) with 10 mg·L^−1^ rifampicin. On day 4 post-hatching, sea bass larvae were incubated one single time with one of the following different treatments: YS0 (*E. coli* with no plasmid), YS1 (*E. coli* expressing truncated DnaK), and YS2 (*E. coli* expressing DnaK). For this procedure, 10^7^ cells/mL suspended in autoclaved seawater (obtained in Section 2.2) were added to each vial. The procedure was conducted in a laminar flow hood to keep the culture under gnotobiotic conditions. As an experimental control of the *E. coli* administration, sea bass larvae incubated with no bacteria (NB group) were also included in the analysis.

### 2.6. Full-Sibling Sea Bass Larvae Incubated with E. coli Overexpressing DnaK and Challenged with Vibrio anguillarum

**Figure 1:**
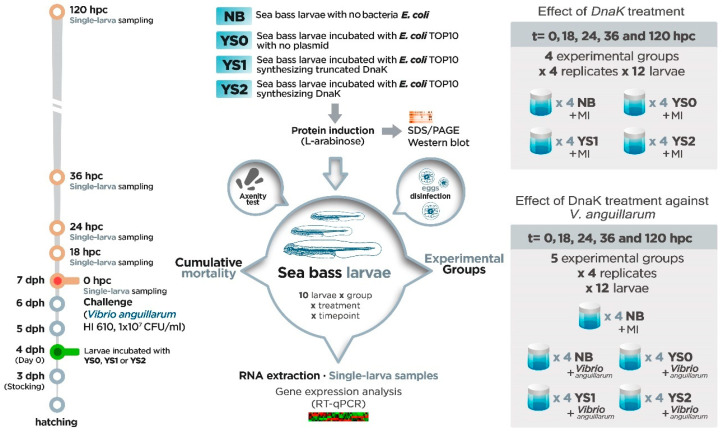
Experimental set-up overview. The timeline shows that on day 3 post-hatching (3 dph), full-sib sea bass larvae were stocked individually in each of the 10 mL sterile screw cap vials (n = 4 replicate vials per treatment; n = 12 larvae each vial). Then, sea bass larvae were incubated at 4 dph one single time with one of the following different treatments: YS0 (*E. coli* with no plasmid), YS1 (*E. coli* expressing truncated DnaK), YS2 (*E. coli* expressing DnaK), and NB (sea bass larvae with no *E. coli*). Three days after incubation (equivalent to 7 dph) with NB, YS0, YS1, or YS2, the effect of DnaK treatment against *V. anguillarum* was evaluated. For this purpose, sea bass larvae were infected with *V. anguillarum* strain HI610 (1 × 10^7^ CFU/mL). Also, the individual effect of treatment was evaluated: for that, all the groups were mock-infected (MI). Samples (12 single larvae × treatment) were collected at 0, 18, 24, 36, and 120 h post-challenge (hpc; orange bold circles in timeline).

Sea bass larvae were bath-incubated with *V. anguillarum* strain HI610 (1 × 10^7^ CFU/mL suspended in autoclaved seawater, as described in Section 2.1) on day three of incubation with *E. coli* overexpressing DnaK (equivalent to 7 dph). The experimental design included the incubation with *V. anguillarum*-free seawater (mock-incubated (MI)) for all the treatments analyzed as an experimental control of the bacterial challenge, including sea bass larvae incubated with no bacteria (NB-MI), *E. coli* with no plasmid (YS0-MI), *E. coli* expressing truncated DnaK (YS1-MI), and *E. coli* expressing DnaK (YS2-MI). For sampling, live larvae were collected at 0, 18, 24, 36, and 120 h post-challenge (hpc). To do this, n = 12 larvae were randomly taken from the four vials assigned to each treatment and immediately stored at −80 °C. The chosen sampling times were determined based on our previous experience analyzing the gene expression profile of sea bass larvae [34]. Survival was monitored in all vials at all sampling points. The experimental set-up overview is shown in Figure 1.

### 2.7. Ethics Statement

The experiment was evaluated and approved by the Ethical Committee of the Faculty of Veterinary Medicine and the Faculty of Bioscience Engineering, Ghent University (no. EC2015_02). The protocol was carried out according to the recommendations of the European Union Ethical Guidelines for experimental animal care and other scientific purposes (2010/63/EU).

### 2.8. RNA Extraction and cDNA Synthesis of Single Full-Sibling Sea Bass Larvae

Total RNA was extracted from single whole larvae with TriReagent following the manufacturer’s instructions. Total RNA concentration was determined by NanoDrop-2000 spectrophotometer (Thermo Scientific, Waltham, MA, USA), and the integrity was measured by Experion RNA StdSens (Bio-Rad). Samples with RNA quality indicator (RQI) values greater than 8 were chosen for gene expression analysis. Five hundred ng of total RNA from each single larva was used to synthesize cDNA with iScript™ cDNA Synthesis Kit (BioRad). The cDNA was used as a template for gene expression analysis.

### 2.9. Gene Expression Analysis by Real-Time PCR

Real-time PCR (Bio-Rad) was performed to analyze the expression pattern of immune-related genes associated with innate immunity (*pentraxin*, *lysozyme*, *hepcidin*, *transferrin*, *hsp70*, *il-1β*, *il-8*, *il-10*, and *ccl4*). The reference candidate genes (*ribosomal protein l13* (*rpl13*), *elongation factor 1α* (*ef1α*), and *40S ribosomal protein SA* (*rpsa*)) were tested using the BestKeeper software [35]. According to previous antecedents on sea bass infected with *V. anguillarum* [34,36], *rpl13* was chosen in our study as a reference gene because of its lower variation tested upon all the samples included in our study. The specific primers used for gene expression analysis are detailed in previous work [34]. The reaction mix (10 μL final volume) consisted of 5 μL of iQ SYBR Green supermix (Bio-Rad), 0.5 μL of forward and reverse primer (500 nM final concentration), 1.5 μL of H_2_O, and 2.5 μL of 1:10 cDNA sample dilution. The running conditions were one step at 95 °C for 3 min, followed by 40 cycles of 10 s at 95 °C and 30 s at 60 °C, following a melting curve dissociation analysis from 65 °C to 95 °C with increments of 0.5 °C. Ten single larvae per time point and treatment were analyzed. Values for each treatment were expressed as normalized relative expression (NRE), calculated in relation to all treatments’ values at time zero, normalized against rpl13 expression following the Pfaffl method, and corrected for the efficiency of each primer set [37]. The results are expressed as the mean value ± standard deviation (SD) for each treatment and time point evaluated.

### 2.10. Statistical Analysis

Cumulative survival curves were compared using the log-rank test corrected for multiple comparisons (*p* < 0.05). Given the rapidity with which the fish of the NB group were dying compared to the rest of the treatments, a Hazard ratio (Mantel-Haenszel test) was carried out. Differences at the gene expression level were evaluated by two-way ANOVA considering the two main effects: treatment and time. Also, the interaction between two factors was considered for the model since we expected differences between groups at different times. Thus, a Tukey’s post-test was performed to test the significant differences between all the groups at each time point included in the study. All the statistical analyses considered a *p* < 0.05 as significant.

## 3. Results

### 3.1. DnaK Induction and Synthesis

**Figure 2:**
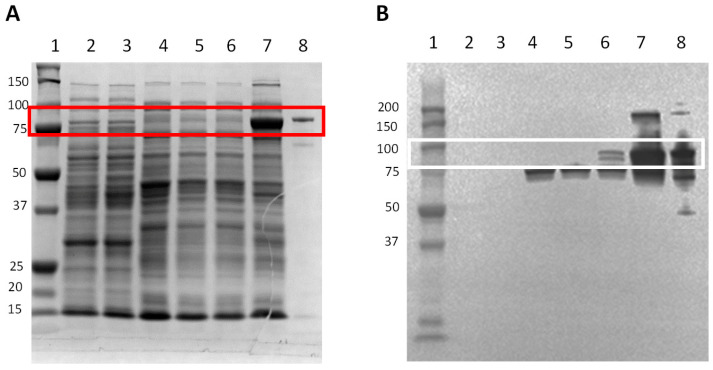
DnaK synthesis induction in cells incubated with ʟ-arabinose. The bacterial cell cultures with an OD_600_ value of 0.5–0.7 were induced with 0.5 mg mL^−1^ of ʟ-arabinose for 4 h. Total bacterial crude protein extract was obtained and was resolved in (**A**) SDS-PAGE stained with Coomassie Biosafe, or (**B**) by Western blot. Fifty micrograms of protein were loaded in each lane. Lane 1: Precision plus protein standard; lane 2: YS0; lane 3: YS0+ ʟ-arabinose; lane 4: YS1; lane 5: YS1+ ʟ-arabinose; lane 6: YS2; lane 7: YS2+ ʟ-arabinose; lane 8: DnaK purified (positive control). Protein molecular weight (kDa) is indicated on the left. Red (SDS-PAGE) and white (WB) boxes show the presence of recombinant DnaK. YS0: *E. coli* TOP10 with no plasmid. YS1: *E. coli* TOP10 expressing truncated DnaK. YS2: *E. coli* TOP10 overexpressing DnaK.

In order to check the DnaK synthesis through the induction by ʟ-arabinose, all the constructs included in this study were incubated with ʟ-arabinose to observe the DnaK synthesis by SDS-PAGE and Western blot (Figure 2). No band associated with DnaK synthesis was observed for YS0 (non-induced; ʟ-arabinose-induced) (Figure 2A, lanes 2 and 3, respectively) and YS1 (Figure 2A, lanes 4 and 5, respectively). In the case of YS2, no band associated with the over-synthesis of DnaK was registered in the non-induced condition (Figure 2A lane 6); by contrast, a positive band representative of the DnaK over-synthesis was observed in the YS2 cell culture induced with ʟ-arabinose (Figure 2A lane 7). The presence and specificity of DnaK on the SDS-PAGE were confirmed by Western blot on the YS2 cell culture incubated with ʟ-arabinose (Figure 2B lane 7). A slight positive band was also noted on the YS2 non-induced cell culture (Figure 2B lane 6). Altogether, these results indicate that the ʟ-arabinose-induced YS2 cell culture over-synthesizes DnaK.

### 3.2. Cumulative Survival in Gnotobiotic Full-Sibling European Sea Bass Larvae Incubated with DnaK

**Figure 3:**
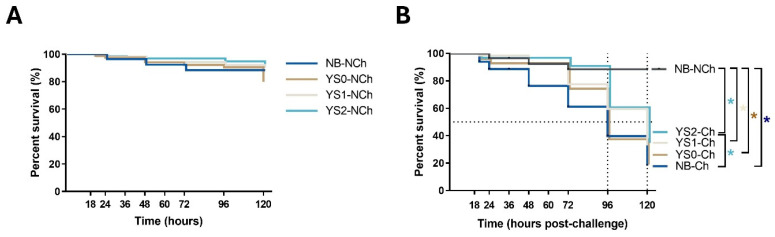
Kaplan-Meier cumulative survival curves of full-sibling European sea bass larvae during 120 h after the pathogen exposure. Larvae were incubated at day 4 post-hatching (YS0, YS1, YS2) and then exposed to *V. anguillarum* on day 7 post-hatching. The graphs represent the cumulative mortality in the first 120 h post-challenge. (**A**) Cumulative survival for non-challenged sea bass larvae. (**B**) Cumulative survival for sea bass larvae challenged with *V. anguillarum*. The time scale indicated in (**A**) represents the same time shown in (**B**). The dotted black line refers to median survival. Asterisk (*) represents the significant difference (*p* < 0.05) in survival (log-rank test corrected for multiple comparisons) compared to control (NB). The line-type details for each group are indicated on the right of each graph, as follows: NB: Sea bass larvae mock-incubated ((**A**): solid line and (**B**): solid black line). YS0: Sea bass larvae incubated with *E. coli* TOP10 with no plasmid (solid brown line). YS1: Sea bass larvae incubated with *E. coli* TOP10 expressing truncated DnaK (solid grey line). YS2: Sea bass larvae incubated with *E. coli* TOP10 overexpressing DnaK (solid light blue line).

In order to evaluate whether DnaK has a protective effect on the survival of full-sibling sea bass larvae challenged with *V. anguillarum*, the cumulative survival of sea bass larvae was recorded after the pathogen exposure (Figure 3). On the non-challenged condition, only slight variations were registered after the mock incubation (Figure 3A). No significant differences were determined between the cumulative survival curves from all the treatments tested during the experiment despite the higher survival trend in fish incubated with DnaK (YS2-MI group). Regarding how rapidly fish are dying, the hazard ratio of YS2-MI compared to the NB-MI group was 1.67 (95% confidence interval [CI], 0.39 to 6.87). These results suggest that DnaK may have a protective effect on sea bass larvae growth under gnotobiotic conditions.

When the sea bass larvae incubated with NB, YS0, YS1, or YS2 were challenged with *V. anguillarum*, a different cumulative survival curve pattern was obtained (Figure 3B). Based on these data, the median survival was determined at 96 hpc for the NB-Ch and YS0-Ch groups and 120 hpc for the YS1-Ch and YS2-Ch groups (Figure 3B, dotted black line). Notably, the cumulative survival curves of YS2-Ch but not YS0-Ch and YS1-Ch were significant compared to the NB-Ch group. This statistical difference was also noted in the rapidity of dying fish of the NB-Ch group compared to YS2-Ch, with a hazard ratio of 2.26 (95% confidence interval [CI], 1.23 to 4.17); meanwhile, a lower ratio was determined for YS1-Ch (2.0; 95% confidence interval [CI], 1.06 to 3.74). These results indicated that DnaK confers protection on gnotobiotic sea bass larvae challenged with *V. anguillarum*.

### 3.3. Innate Immune-Related Gene Expression Profile in Gnotobiotic Full-Sibling European Sea Bass Larvae Incubated with DnaK

**Figure 4:**
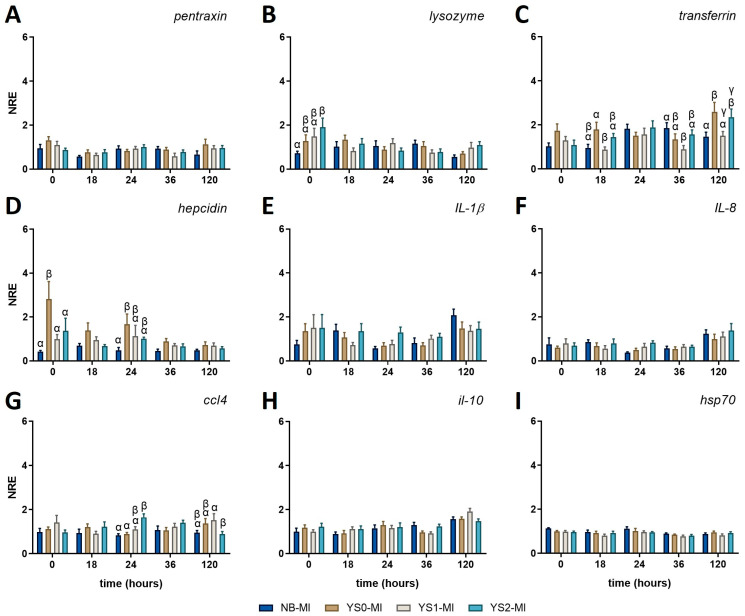
Normalized relative expression (NRE) of innate immune-related genes in full-sibling European sea bass larvae treated with DnaK. The graphs represent the normalized relative expression (NRE; mean value ± SD) for (**A**) *pentraxin*; (**B**) *lysozyme*; (**C**) *transferrin*; (**D**) *hepcidin*; (**E**) *interleukin* (*il*)*-1β*; (**F**) *il-8*; (**G**) *CC motif chemokine 4* (*ccl4*); (**H**) *il-10*; and (**I**) *heat shock protein 70* (*hsp70*). The obtained values were statistically analyzed by two-way ANOVA and Tukey’s post-test for significant differences (*p*-value < 0.05). The statistical difference between the control group (NB-MI) and YS0-MI, YS1-MI, or YS2-MI at each time point analyzed are indicated by different Greek letters. NB-MI: sea bass larvae incubated with no bacteria and Mock Infected (NB-MI group; blue bar). YS0-MI: Sea bass larvae incubated with *E. coli* TOP10 with no plasmid and Mock Infected (brown bar). YS1-MI: Sea bass larvae incubated with *E. coli* TOP10 expressing truncated DnaK and Mock Infected (fawn bar). YS2-MI: Sea bass larvae incubated with *E. coli* TOP10 expressing DnaK and Mock Infected (light blue bar).

The expression profile of several genes related to innate immunity was conducted in non-challenged fish to determine whether the administration of DnaK had a differential modulatory effect on sea bass larvae. For this purpose, DnaK was administered to sea bass larvae at day 4 post-hatching. Then, the expression profile was assessed three days after the DnaK administration (equivalent to 7 days post-hatching and indicated as zero hours post-challenge, 0 hpc). In the case of non-challenged fish (MI), no variations were registered for the expression of *pentraxin* (Figure 4A), *il-1β* (Figure 4E), il-8 (Figure 4F), *il-10* (Figure 4H), and *hsp70* (Figure 4I) at any of the time points assessed. For the other innate-related genes, only punctual variations were registered. In this way, the treatment with DnaK promoted the upregulation of *lysozyme* at 0 hpc in the gnotobiotic sea bass larvae treated with YS2-MI compared to the NB-MI group (Figure 4B), to then return its value to the basal level. On the other hand, the expression of *transferrin* (involved in removing iron from the bloodstream) showed the downregulation of YS1-MI at 18 hpc and 120 hpc compared to YS0-MI (Figure 4C). In the same way, the expression of *transferrin* for YS1-MI was also downregulated at 36 hpc. Importantly, the upregulation of *transferrin* was identified for YS2-MI at 120 hpc compared to the NB-MI group (Figure 4C). A similar effect was also observed for YS0-MI (Figure 4C).

*Hepcidin* modulation (the principal circulating regulator of iron absorption and distribution across tissues) was also noted, registering an increase at 0 hpc in the YS0-MI group compared with all the rest of the conditions (Figure 4D). In the same line, the *hepcidin* expression for YS0-MI at 24 hpc was also upregulated compared with the NB-MI group.

The expression of the inflammatory chemokine *ccl4* (also known as macrophage inflammatory protein-1β [MIP-1β]) was upregulated at 24 h in YS2-MI compared to NB-MI and YS0-MI groups. Then, the expression for YS2-MI was downregulated compared to YS1-MI, but no difference was observed compared to the NB-MI group (Figure 4G). The NRE (mean ± SD) for all genes evaluated and the *p*-values obtained for each comparison can be found in Table A1 and Table A2, respectively.

Collectively, our results suggest that DnaK does not activate a robust immune response in gnotobiotic sea bass larvae but seems to promote bacteriolytic, iron regulation, and chemotaxis.

### 3.4. Innate Immune-Related Gene Expression Profile in Gnotobiotic Full-Sibling European Sea Bass Larvae Incubated with DnaK and Challenged with V. anguillarum

**Figure 5:**
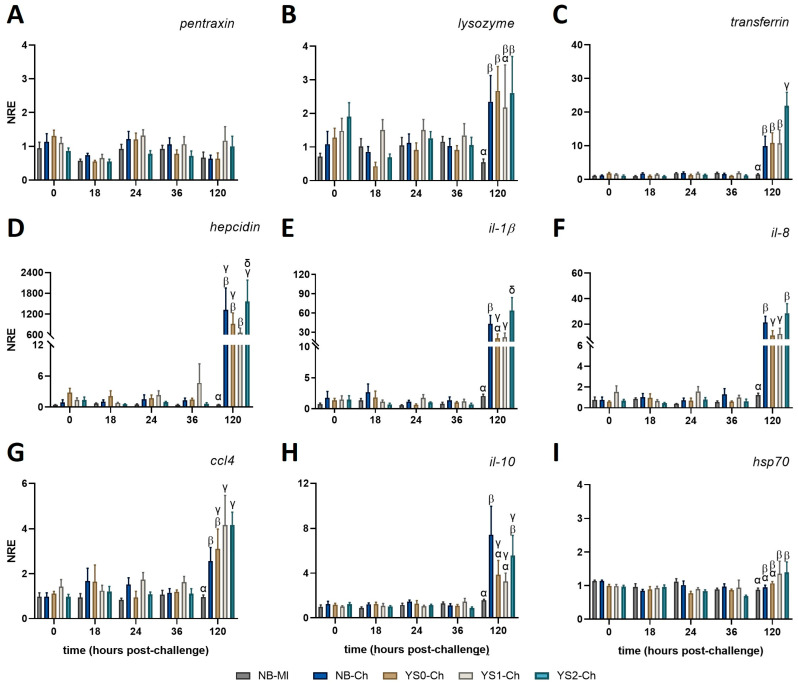
Normalized relative expression of innate immune-related genes in full-sibling European sea bass larvae treated with DnaK and challenged with *V. anguillarum*. Larvae were incubated at day 4 post-hatching and then exposed to *V. anguillarum* at day 7 post-hatching. The graphs represent the normalized relative expression (NRE; mean value ± SD) for (**A**) *pentraxin*; (**B**) *lysozyme*; (**C**) *transferrin*; (**D**) *hepcidin*; (**E**) *interleukin* (*il*)*-1β*; (**F**) *il-8*; (**G**) *CC motif chemokine 4* (*ccl4*); (**H**) *il-10*; and (**I**) *heat shock protein 70* (*hsp70*). The obtained values were evaluated by two-way ANOVA and Tukey’s post-test for significant differences (*p* < 0.05). Different Greek letters indicate statistical differences between the control group (NB-MI) and NB-Ch, YS0-Ch, YS1-Ch, or YS2-Ch at each time point analyzed. NB: Sea bass larvae incubated with no bacteria (NB group; mock-incubated; grey and blue bar). YS0: Sea bass larvae incubated with *E. coli* TOP10 with no plasmid (brown bar). YS1: Sea bass larvae incubated with *E. coli* TOP10 expressing truncated DnaK (fawn bar). YS2: Sea bass larvae incubated with *E. coli* TOP10 expressing DnaK (light blue bar).

In order to evaluate whether the administration of DnaK modulated the response of sea bass larvae challenged with *V. anguillarum*, we analyzed the expression pattern of a set of genes related to innate immunity. No variations were observed in *pentraxin* at any of the time points evaluated (Figure 5A). All the other genes evaluated showed at least one significant upregulation at 120 hpc in the challenged groups (NB-Ch; YS0-Ch; YS1-Ch; YS2-Ch) compared with the mock-incubated group (NB-MI) (Figure 5). Importantly, YS2-Ch was the only experimental group that showed a significant upregulation in all the genes assessed, suggesting the role of DnaK in promoting defensive mechanisms in sea bass against *V. anguillarum* (Figure 5). In comparing all time points evaluated, an upregulation of *lysozyme* expression was observed at 120 h post-challenge (hpc) in all the challenged groups except YS1-Ch compared to the NB-MI group (Figure 5B).

The upregulation in the expression of *transferrin* was observed at 120 h post-challenge (hpc) in larvae incubated with YS2-Ch (Figure 5C) compared with all other treatments (NB-MI; NB-Ch; YS0-Ch; YS1-Ch). A notorious marked increase in *hepcidin* expression compared with the NB-MI group was observed at 120 hpc in all the treatments challenged with *V. anguillarum* (Figure 5D). However, only a significant upregulation of *hepcidin* was identified in sea bass larvae incubated with YS2-Ch compared with the YS1-Ch group (Figure 5D).

A similar expression pattern was observed at the pro-inflammatory level. Thus, the expression of *il-1β* (Figure 5E) and *il-8* (Figure 5F) at 120 hpc showed their upregulation in fish incubated with YS2-Ch compared with YS0-Ch, YS1-Ch, and NB-Ch groups. An increase in the expression of *il-8* was observed in all treatments challenged with *V. anguillarum* compared with the NB-MI group at 120 hpc (Figure 5F). Importantly, the expression of both cytokines was downregulated in the YS0-Ch and YS1-Ch treatments compared with the NB-Ch group. The expression of *ccl4* was upregulated in all the challenged groups, even showing a significant increase in their expression for YS1-Ch and YS2 compared to the NB-Ch group. Despite these differences, no significant differences were registered between YS2-Ch and YS1-Ch or YS0-Ch groups. However, an increase in *ccl4* expression was observed in all treatments compared to the NB-MI group at 120 hpc (Figure 5G).

The anti-inflammatory cytokine *il-10* showed a higher expression in NB-Ch at 120 hpc compared to YS0-Ch and YS1-Ch. On the other hand, the YS2-Ch group showed an augment in its expression profile compared with the NB-MI group, but no differences compared with the other challenged treatments (Figure 5H). None of the treatments in the study modified the expression of *hsp70*, although an increase was noted for the YS1-Ch and YS2-Ch compared to the NB-MI group (Figure 5I). The gene expression data (mean ± SD) for all evaluated genes and the *p*-values obtained for each comparison in full-sibling European sea bass larvae treated with DnaK and challenged with *V. anguillarum* can be found in Table A3 and Table A4, respectively.

Taken together, these results showed that sea bass larvae incubated with DnaK had an increased modulatory effect on genes related to iron regulation and pro-inflammatory cytokines at 120 hpc, which is related to the observed protection of DnaK on larval survival at 120 hpc.

## 4. Discussion

Several stress factors affect fish in the aquatic environment. It has been widely described that stress and immune response are closely connected, in which a stressor can induce alterations in innate immune responses. These stress factors can make fish more sensitive to pathogen attacks, such as *Vibrio anguillarum* [14,38]. This bacterium generates major problems in aquaculture due to high mortality, causing significant economic losses [34]. Therefore, studying the interaction between the nervous, endocrine, and immune systems is essential to allow the survival of these fish [14].

Bacterial endocytic shock proteins, such as DnaK, stand out for their ability as potent immunomodulators and present considerable potential as therapeutic agents in infection control in aquaculture, as documented in previous studies [21,39]. This effect is partly due to their presence both in the cytoplasm and on the surface of bacteria, facilitating their delivery to larvae by making them more accessible [40]. Thus, feeding with DnaK-enriched bacteria to aquatic organisms emerges as a novel strategy in disease management in aquaculture [1,41]. The advantages of this strategy are that using bacteria that can autonomously produce and secrete DnaK makes it more sustainable, efficient, and economical in the long term compared to direct protein production and administration. Thus, the novelty of this work lies in a different approach consisting of providing DnaK to gnotobiotic sea bass larvae. However, one limitation of the study is the absence of a direct comparison between the *E. coli*-based delivery system and the conventional encapsulated microparticle approach previously used in sea bass larvae [20]. The analysis of this comparison in future studies will provide valuable insight into the relative efficacy of both methods. Prospectively, the scalability and applicability of this novel strategy in aquaculture farms should be explored and analyzed in future studies.

In this study, we observed that sea bass larvae presented high mortality *against V. anguillarum* infection. This effect is probably attributable to fish larvae’s defense relying specifically on their innate immune system, as their adaptive immune system is not yet sufficiently developed [34,42]. Consequently, larvae are more vulnerable to pathogens present in the aquatic environment than adults, leading to high mortality rates [42]. Also, during the first two weeks after hatching, sea bass larvae in gnotobiotic conditions do not present mucus-producing cells (goblet cells), so they do not present this physical barrier in the gut, remaining exposed [43]. In fact, previous works have found high numbers of *V. anguillarum* in the gastrointestinal tract of gnotobiotic sea bass larvae when exposed to the bacterium [43]. In our study, we observed that gnotobiotic sea bass larvae incubated with DnaK showed a higher survival against *V. anguillarum* infection than larvae not incubated with this protein. These results agree with previous studies that demonstrated that *V. anguillarum* induces a gradual decrease in larval survival of sea bass under gnotobiotic conditions and that DnaK has a protective role in the survival of aquatic organisms [31,34]. Importantly, significant differences in the expression of genes related to pro-inflammatory (*lysozyme*, *transferrin*, *hepcidin*) and antimicrobial (*il-1β*, *il-8*, *ccl4*, *il-10*) response in larvae incubated with DnaK also occurred at 120 hpc. These antecedents are consistent with the significant difference in cumulative survival mentioned above. Taken together, we can infer that DnaK has a protective effect on the growth of sea bass larvae under gnotobiotic conditions against pathogen infection by activating the expression of different genes related to the innate immune response.

The heat shock protein 70 is involved in many cellular processes, including synthesis, translocation, proper folding, and degradation of proteins. In addition, it participates in antigen presentation and activation of immune cells such as lymphocytes and dendritic cells. In fish, HSP70 is induced by stress factors such as high temperatures, microbial infections, and heavy metals [14]. It has been described in teleost fish larvae that HSP70 protein expression can be used as a marker of larval stress [25]. Regarding cell stress response, this protein plays a vital role in cortisol binding to the glucocorticoid receptor (GR), considered the primary receptor for glucocorticoid activity in teleost fish. In this regard, a correlation between the positive regulation of glucocorticoid receptors (*gr1* and *gr2*) and *hsp70* gene expression has been described [34]. An unexpected finding was that *hsp70* expression was not upregulated at any time point analyzed after infection with *V. anguillarum*. This differs from the results obtained by Dang et al. (2010) [7], who reported that infection with *V. anguillarum* increased *hsp70* expression in rainbow trout (*Oncorhynchus mykiss*). However, basal *hsp70* expression has been reported in gnotobiotic sea bass larvae even after exposure to this bacterium [34]. These results are consistent with other research indicating that increased expression of HSP70 is only evident in 40-day-old stressed sea bass larvae [14]. Furthermore, several studies have specified that cortisol may not be associated with increased gr or *hsp70* expression [44,45]. This background would indicate that cortisol does not modulate *hsp70* expression in gnotobiotic sea bass larvae challenged with *V. anguillarum* at these early stages of larval development [34].

It has been reported that fish rely mainly on innate immune defense in their first days after hatching (64–68 days). Such a response is crucial in the early fight against infections by pathogenic microorganisms. Also, previous studies in fish have described that, at hatching and subsequent weeks, the expression of components with antimicrobial activity associated with the humoral arm of innate immunity (*pentraxin*, *lysozyme*, *transferrin*, *hepcidin*) is induced [34,46]. This background suggests that these molecules play a role in preparing the organism against potential environmental pathogens that may be encountered during the early stage of larval development. Consequently, the modulation of genes associated with innate immunity in the response of sea bass larvae against *V. anguillarum* is of great importance [34].

Like in mammals, fish can produce acute phase proteins (APPs) during infection or because of the presence of danger or stress signals, being one of the most common pentraxins. These APPs activate crucial signaling cascades to induce a pro-inflammatory response [34,47]. Pentraxins are phylogenetically conserved soluble pattern recognition receptors (PRRs) that are associated with the acute phase response (APR) and mediate agglutination, complement system activation, and opsonization [34,47]. Our study found no variation in pentraxin gene expression in both bacterially challenged and unchallenged larvae. These results are in line with previous studies, which described that sea bass larvae under gnotobiotic conditions do not positively regulate pentraxin expression after infection with *V. anguillarum* [34], unlike other teleost fish such as *Oncorhynchus mykiss* [48], *Plecoglossus altivelis* [49] and *Gadus morhua* [50]. Thus, the non-modulatory response of pentraxin in sea bass larvae in response to the incubation with *V. anguillarum* suggests that other receptors and molecules of innate immunity might be involved in the recognition and response against this pathogenic bacterium [34]. Furthermore, these results suggest that administration with DnaK does not modulate pentraxin gene expression pathways at times analyzed. Consequently, gene expression analysis should be conducted at more time points to support this hypothesis.

Lysozyme is a vital immune system bacteriolytic enzyme with a broad bactericidal spectrum. It cleaves the bacterial wall by hydrolyzing the β-[1,4]-glucosidic linkage of peptidoglycans [29,34]. This study found that uninfected fish incubated with YS2 presented a significant increase in *lysozyme* expression at 0 h (equivalent to 7 dph). Meanwhile, larvae incubated with YS0 and YS1 maintained a constant basal expression of this enzyme compared to other conditions. These findings are in agreement with those described by Cecchini et al. (2000) [51], who indicated that the expression of *lysozyme* in sea bass (*Dicentrarchus labrax*) larvae is observed up to 24 h after hatching, experiencing a significant decrease in its expression after that time. The results also showed an increase in *lysozyme* expression at 120 hpc, with no effect on the expression of this enzyme in larvae incubated with DnaK. This suggests that sea bass larvae would not recognize this bacterium early during the first 36 hpc [34].

Transferrin and hepcidin are another group of acute-phase proteins involved in the innate immune response and the regulation of iron homeostasis. Transferrin is a plasma iron-binding glycoprotein that controls free iron levels in biological fluids. This glycoprotein has bacteriostatic activity by chelating available iron to make it inaccessible to invading pathogenic bacteria [30,34]. Hepcidin is an antimicrobial peptide (AMP) with a wide range of actions. In mammals, it has been described to be involved in the body’s defense against pathogens, being considered a significant player in the innate immune system. Hepcidin has been identified in several teleost fish species, including European sea bass (*Dicentrarchus labrax*), Atlantic salmon (*Salmo salar*), and rainbow trout (*Oncorhynchus mykiss*), among others. Its structure is conserved in these species, suggesting its participation in defense responses in fish against pathogenic infections [52].

An interesting finding of our study is that gnotobiotic sea bass larvae incubated with DnaK presented at 120 hpc a significant overexpression of *transferrin* and *hepcidin* against *V. anguillarum* infection. Such a response would promote antimicrobial activity (driven by *hepcidin*) and the regulation and restriction of iron in the bloodstream by transferrin, thus reducing its bioavailability for *V. anguillarum* growth. These results are related to previous studies in sea bass, which indicate that synthetic *hepcidin* has an inhibitory effect on the growth of this bacterium and presents elevated expression levels when stimulated with DnaK [20,52]. Iron is crucial for bacteria to grow and achieve infection, so the ability to compete for iron availability in the bloodstream is crucial in establishing the ability of the fish to overcome pathogen infection. Taken together, these data suggest that sea bass larvae incubated with DnaK have enhanced survival by favoring an antimicrobial environment against *V. anguillarum* infection.

The strategy of delivering immune molecules in expression vectors has been used previously. Various strategies have been used, including the administration of proinflammatory cytokines (IL-β, IL-6, TNFα, IL-8, IL-22, IFN-1, IL-17C) [53,54,55,56,57], activators of innate immunity (C-type lectin, PGRP, NCCRP) [58,59], transcription factors (IRF3) [60] in different fish species. Thus, the administration of immune-related molecules improves the health status of fish and protects them against bacterial pathogens [60] by stimulating the expression of proinflammatory molecules. Precisely, a key mechanism in initiating antibacterial responses is the co-expression of pro-inflammatory cytokines, such as IL-1β, IL-8 and CCL4 [34]. Importantly, in our study, we observed a positive upregulation at 120 hpc of the previously mentioned innate effectors, which are directly associated with the positive upregulation of *il-1β*, *il-8*, and *ccl4*. IL-1β is a cytokine released in the early stage of response against infection and initiates pro-inflammatory responses, allowing the synthesis of other cytokines and the activation of different immune cells. In teleost fish, *il-1β* expression in stressed fish has been reported to increase up to 8-fold [3]. Chistiakov et al. (2010) [61] demonstrated that *il-1β* is involved in the resistance process of European sea bass against *V. anguillarum*.

Moreover, in the context of DnaK administration, it has been described that this protein is recognized as an immunodominant antigen that induces a strong humoral and cellular immune response in fish. This response appears to activate Toll-like receptors (TLR2, TLR4) that induce pro-inflammatory signals and promote resistance against disease [22,31]. Positive regulation of *il-1β* is also accompanied by the expression of *il-8* and increased expression of *ccl4*. IL-8 and CCL4 are chemokines produced by macrophages that have chemoattractant activity for neutrophils and T cells at sites of infection [34]. Therefore, the increased survival of sea bass larvae against this bacterium observed up to 120 hpc suggests that DnaK promotes elevated expression of these pro-inflammatory cytokines and chemoattractants that favor the activation of an antimicrobial response.

The pro-inflammatory response is controlled by anti-inflammatory cytokines that regulate its expression and promote tissue repair. IL-10 is an anti-inflammatory cytokine that contributes to the resolution of pathogen-mediated inflammation and the reduction of tissue damage caused by inflammation [34]. This study observed a higher value of normalized relative expression at 120 hpc in the NB-Ch and incubated groups with DnaK (YS2-Ch). On the other hand, the groups incubated only with plasmid (YS0-Ch) and YS1-Ch (truncated DnaK) showed underexpression compared to the NB-Ch group. The higher expression of *il-10* in the NB-Ch group would suggest that *V. anguillarum* infection promotes the control of the pro-inflammatory response in sea bass larvae. This relates to previous studies indicating that this bacterium can evade the fish’s immune system by inhibiting the respiratory burst of M1 macrophages [20]. Indeed, this result agrees with previous studies in untreated sea bass larvae in which an increase in *il-10* expression at 120 hpc was identified in sea bass larvae challenged with *V. anguillarum* [34]. Interestingly, such an increase would be linked to the physiological stress response mounted by the fish and the increase in glucocorticoids at 120 hpc, and the induction of a systemic anti-inflammatory environment [34]. On the other hand, the increased value of *il-10* (although not significant) in the YS2-Ch group would suggest that the activation of mechanisms controlling the pro-inflammatory response could take place in sea bass larvae challenged with *V. anguillarum.* This non-significant but increased effect on *il-10* expression could be due to a temporal effect and the times chosen for the analysis, as suggested for the modulation of immune response-associated genes reported in other studies [62]. Apparently, promoting control of the pro-inflammatory response could also be linked to increased survival in sea bass larvae challenged with *V. anguillarum* by regulating inflammation and activating eventual tissue damage mechanisms [3].

**Figure 6:**
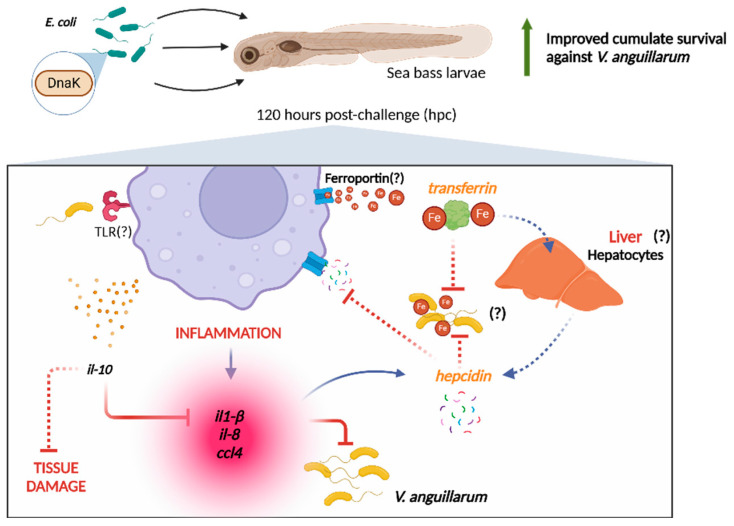
Summary of the effects promoted by the administration of DnaK to gnotobiotic sea bass larvae challenged with *Vibrio anguillarum*. The administration of DnaK modulates the expression of pro-inflammatory (*il-1β*, *IL-8*, *CCL4*) and anti-inflammatory (*IL-10*) cytokines in gnotobiotic sea larvae after 120 hpc with *V. anguillarum*. We hypothesize that TLR triggers these mechanisms in response to recognizing *V. anguillarum* pathogen-associated molecular patterns (PAMPs). These pro-inflammatory cytokines stimulate hepcidin production. Hepcidin inhibits ferroportin, the principal iron transporter, limiting the iron availability in the environment, while *transferrin* restricts the remaining iron. The action of both proteins prevents iron uptake by *V. anguillarum*, influencing its proliferation. The question marks and dashed lines represent topics we propose based on our results, but need validation in future studies. Figure created with Biorender (https://biorender.com).

We hypothesize that the delay in activating the immune response promoted by the pro-inflammatory and antimicrobial genes involved in the response against *V. anguillarum* may have contributed to the high mortality observed up to 120 hpc. This delay in the response could have caused a delay in the processes necessary to sequester available iron from the bloodstream, thereby inhibiting this bacterium’s growth. This may be due to the fact that *V. anguillarum* uses iron acquisition mechanisms for its growth even if transferrin is present in fish [20]. Likewise, it has been described that this pathogenic bacterium has the ability to evade the immune system of sea bass by interfering negatively with leukocytes, thus explaining the delayed response to this pathogen even with DnaK pretreatment [63,64]. Despite these antecedents, larvae incubated with DnaK before being challenged with *V. anguillarum* showed increased expression at 120 hpc of a set of genes involved in the organism’s defense. This immune response activation suggests that DnaK administration induces a potent innate immune response that enhances the survival of sea bass larvae challenged with *V. anguillarum*. We highlight that this response takes place in an organism even before the full maturation of the immunological system in fish larvae, thus reinforcing the immunoprotective role of DnaK against pathogenic infections in sea bass larvae. A graphical summary of our main results describing the modulatory effect of DnaK on sea bass larvae is shown in Figure 6.

## 5. Conclusions

This study describes the effect of DnaK administration in modulating the immune response in gnotobiotic sea bass larvae challenged with *V. anguillarum*, demonstrating its potential as a pathogen resistance agent in fish by improving fish survival. The results showed a significant upregulation of antimicrobial (*transferrin*, *hepcidin*) and proinflammatory (*il-1β*, *il-8*, *ccl4*) genes after DnaK administration at 120 hpc, despite the underdeveloped immune system of the larvae. This strategy would enable improved disease management in aquaculture farms; however, future studies are needed to evaluate its efficacy on other pathogens and in different fish species.

## Data Availability

The original contributions presented in the study are included in the article/Appendix A. Further inquiries can be directed to the corresponding author.

## Appendix A

**Table A1:** Normalized relative expression (NRE) values of innate immune-related genes in full-sibling European sea bass larvae treated with DnaK at different time points (0, 18, 24, 36, and 120 h).

Genes	Time (hpc)	NB-MI		YS0-MI		YS1-MI		YS2-MI	
		Mean	SD	Mean	SD	Mean	SD	Mean	SD
*pentraxin*	0	0.94	0.48	1.31	0.53	1.10	0.51	0.87	0.25
	18	0.57	0.16	0.76	0.35	0.64	0.24	0.77	0.35
	24	0.93	0.42	0.82	0.24	0.93	0.34	1.01	0.33
	36	0.93	0.30	0.89	0.31	0.59	0.43	0.78	0.31
	120	0.67	0.50	1.13	0.68	0.94	0.36	0.97	0.34
*lysozyme*	0	0.71	0.26	1.27	0.90	1.48	1.17	1.90	1.24
	18	1.02	0.74	1.34	0.63	0.82	0.46	1.16	0.67
	24	1.05	0.66	0.89	0.41	1.18	0.64	0.84	0.35
	36	1.16	0.46	1.06	0.59	0.75	0.40	0.77	0.45
	120	0.55	0.29	0.71	0.26	0.98	0.64	1.10	0.46
*trasnsferrin*	0	1.03	0.41	1.74	0.97	1.30	0.54	1.10	0.65
	18	0.96	0.50	1.79	1.00	0.88	0.38	1.46	0.47
	24	1.83	0.64	1.51	0.50	1.57	0.89	1.89	0.93
	36	1.85	0.75	1.34	0.79	0.89	0.54	1.57	0.63
	120	1.47	0.63	2.60	1.26	1.51	0.55	2.36	1.15
*hepcidin*	0	0.43	0.14	2.82	2.52	0.99	0.61	1.37	1.73
	18	0.69	0.34	1.39	1.02	0.95	0.48	0.68	0.19
	24	0.48	0.40	1.68	1.45	1.13	1.54	1.01	0.21
	36	0.45	0.26	0.89	0.42	0.71	0.25	0.67	0.35
	120	0.48	0.17	0.72	0.45	0.69	0.36	0.57	0.24
*il-1β*	0	0.75	0.51	1.36	1.05	1.50	1.69	1.51	1.80
	18	1.38	0.86	1.07	0.68	0.73	0.35	1.35	1.03
	24	0.57	0.24	0.70	0.41	0.77	0.54	1.30	0.75
	36	0.81	0.69	0.71	0.37	1.02	0.47	1.10	0.47
	120	2.08	0.89	1.48	0.88	1.37	0.68	1.46	0.96
*il-8*	0	0.76	0.77	0.61	0.20	0.79	0.57	0.69	0.38
	18	0.85	0.33	0.67	0.46	0.56	0.42	0.79	0.62
	24	0.38	0.08	0.49	0.25	0.64	0.50	0.82	0.29
	36	0.57	0.30	0.54	0.31	0.65	0.29	0.64	0.22
	120	1.23	0.58	0.99	0.68	1.12	0.53	1.39	0.98
*ccl4*	0	0.98	0.49	1.11	0.31	1.42	1.00	0.97	0.32
	18	0.94	0.51	1.20	0.45	0.90	0.35	1.23	0.64
	24	0.83	0.25	0.89	0.24	1.11	0.38	1.65	0.48
	36	1.07	0.57	1.06	0.40	1.22	0.51	1.40	0.39
	120	0.95	0.32	1.38	0.64	1.53	0.83	0.89	0.37
*il-10*	0	1.00	0.42	1.17	0.40	1.00	0.25	1.22	0.46
	18	0.89	0.30	0.92	0.40	1.11	0.34	1.12	0.41
	24	1.15	0.45	1.29	0.54	1.17	0.37	1.21	0.57
	36	1.29	0.38	0.96	0.20	0.92	0.25	1.24	0.31
	120	1.57	0.31	1.59	0.24	1.92	0.38	1.48	0.33
*hsp70*	0	1.14	0.08	0.99	0.14	0.99	0.15	0.97	0.13
	18	0.96	0.30	0.92	0.26	0.81	0.21	0.93	0.22
	24	1.12	0.27	1.02	0.38	0.97	0.18	0.96	0.08
	36	0.89	0.11	0.85	0.11	0.77	0.19	0.80	0.16
	120	0.87	0.18	0.95	0.20	0.81	0.20	0.93	0.18

**Table A2:** Summary of statistical differences (*p* values) from Tuckey’s multiple comparison test for the relative expression of different genes evaluated in sea bass larvae treated with DnaK at different time points (0, 18, 24, 36, and 120 h). Significant differences (*p* < 0.05) are highlighted in bold.

Genes	Group Comparison	Hours Post Administration of DnaK				
		0	18	24	36	120
*pentraxin*	NB-MI vs. YS0-MI	0.2129	0.7074	0.9298	0.996	0.0506
	NB-MI vs. YS1-MI	0.8373	0.9732	>0.9999	0.2203	0.4512
	NB-MI vs. YS2-MI	0.9816	0.6739	0.9636	0.8262	0.3233
	YS0-MI vs. YS1-MI	0.6127	0.9144	0.9286	0.3008	0.7478
	YS0-MI vs. YS2-MI	0.0633	>0.9999	0.6987	0.9146	0.7897
	YS1-MI vs. YS2-MI	0.5575	0.894	0.9644	0.6908	0.9992
*lysozyme*	NB-MI vs. YS0-MI	0.29	0.6633	0.9493	0.9864	0.9529
	NB-MI vs. YS1-MI	0.0751	0.903	0.9741	0.5091	0.4884
	NB-MI vs. YS2-MI	**0.0018**	0.959	0.9051	0.561	0.2287
	YS0-MI vs. YS1-MI	0.8867	0.2641	0.736	0.705	0.81
	YS0-MI vs. YS2-MI	0.1457	0.9278	0.9988	0.7548	0.5441
	YS1-MI vs. YS2-MI	0.4821	0.6493	0.646	0.9998	0.9815
*transferrin*	NB-MI vs. YS0-MI	0.2237	0.0818	0.7765	0.4656	**0.0074**
	NB-MI vs. YS1-MI	0.8907	0.9952	0.8722	**0.0327**	0.9996
	NB-MI vs. YS2-MI	0.9977	0.4819	0.9978	0.8574	**0.0458**
	YS0-MI vs. YS1-MI	0.5808	**0.0459**	0.9975	0.5429	**0.0174**
	YS0-MI vs. YS2-MI	0.2523	0.7831	0.668	0.9177	0.8982
	YS1-MI vs. YS2-MI	0.9423	0.3465	0.7815	0.2148	0.086
*hepcidin*	NB-MI vs. YS0-MI	**<0.0001**	0.358	**0.0289**	0.7354	0.943
	NB-MI vs. YS1-MI	0.6525	0.9251	0.4246	0.9286	0.963
	NB-MI vs. YS2-MI	0.1929	>0.9999	0.5981	0.9567	0.9958
	YS0-MI vs. YS1-MI	**0.0003**	0.7279	0.5578	0.9741	>0.9999
	YS0-MI vs. YS2-MI	**0.0047**	0.3662	0.3834	0.952	0.9864
	YS1-MI vs. YS2-MI	0.8373	0.9215	0.9918	0.9996	0.9934
*il-1β*	NB-MI vs. YS0-MI	0.4449	0.8649	0.9901	0.9953	0.4291
	NB-MI vs. YS1-MI	0.2986	0.3479	0.9613	0.9532	0.2998
	NB-MI vs. YS2-MI	0.2632	0.9999	0.2538	0.8965	0.3791
	YS0-MI vs. YS1-MI	0.9841	0.8235	0.9981	0.8582	0.9926
	YS0-MI vs. YS2-MI	0.979	0.8928	0.4181	0.7685	>0.9999
	YS1-MI vs. YS2-MI	>0.9999	0.3856	0.5025	0.9971	0.9951
*il-8*	NB-MI vs. YS0-MI	0.9271	0.8564	0.9605	0.9995	0.6896
	NB-MI vs. YS1-MI	0.9988	0.5566	0.6531	0.9833	0.9597
	NB-MI vs. YS2-MI	0.9925	0.9936	0.1957	0.9866	0.8876
	YS0-MI vs. YS1-MI	0.8659	0.9599	0.9029	0.9618	0.9448
	YS0-MI vs. YS2-MI	0.9862	0.9509	0.417	0.9675	0.2748
	YS1-MI vs. YS2-MI	0.9728	0.7245	0.8285	>0.9999	0.6411
*ccl4*	NB-MI vs. YS0-MI	0.9366	0.6931	0.9942	>0.9999	0.2572
	NB-MI vs. YS1-MI	0.2473	0.9985	0.6058	0.9154	0.0809
	NB-MI vs. YS2-MI	>0.9999	0.6155	**0.0019**	0.4821	0.9932
	YS0-MI vs. YS1-MI	0.5279	0.5916	0.7607	0.9016	0.9317
	YS0-MI vs. YS2-MI	0.9252	0.9993	**0.0047**	0.446	0.1559
	YS1-MI vs. YS2-MI	0.2144	0.5129	0.0776	0.8535	**0.0429**
*il-10*	NB-MI vs. YS0-MI	0.7923	0.9984	0.8448	0.2293	0.9995
	NB-MI vs. YS1-MI	>0.9999	0.5506	0.9998	0.1309	0.199
	NB-MI vs. YS2-MI	0.6378	0.5435	0.9834	0.9901	0.9423
	YS0-MI vs. YS1-MI	0.7655	0.677	0.8742	0.9916	0.2638
	YS0-MI vs. YS2-MI	0.9924	0.6665	0.9658	0.3563	0.9111
	YS1-MI vs. YS2-MI	0.5956	>0.9999	0.9917	0.2178	0.0613
*hsp70*	NB-MI vs. YS0-MI	0.4408	0.9584	0.6403	0.9663	0.8582
	NB-MI vs. YS1-MI	0.4316	0.3345	0.3182	0.5489	0.9171
	NB-MI vs. YS2-MI	0.3211	0.9805	0.2748	0.8029	0.9326
	YS0-MI vs. YS1-MI	>0.9999	0.6666	0.95	0.8145	0.5146
	YS0-MI vs. YS2-MI	0.9936	0.9995	0.9241	0.9697	0.9967
	YS1-MI vs. YS2-MI	0.9947	0.5926	0.9998	0.9729	0.622

**Table A3:** Normalized relative expression values of innate immune-related genes in full-sibling European sea bass larvae treated with DnaK and challenged with *V. anguillarum* at different time points (0, 18, 24, 36, and 120 h).

Genes	Time (hpc)	NB-Ch		YS0-Ch		YS1-Ch		YS2-Ch	
		Mean	SD	Mean	SD	Mean	SD	Mean	SD
*pentraxin*	0	1.13	0.69	1.31	0.53	1.10	0.51	0.87	0.25
	18	0.74	0.16	0.55	0.10	0.66	0.32	0.55	0.21
	24	1.22	0.64	1.21	0.52	1.32	0.55	0.78	0.28
	36	1.06	0.55	0.78	0.35	1.07	0.69	0.72	0.44
	120	0.63	0.32	0.63	0.41	1.17	1.09	1.00	0.89
*lysozyme*	0	1.08	1.08	1.27	0.90	1.48	1.17	1.90	1.24
	18	0.85	0.47	0.43	0.34	1.51	0.90	0.69	0.29
	24	1.12	0.75	0.91	0.59	1.51	0.99	1.26	0.62
	36	1.03	0.62	0.92	0.39	1.35	1.10	1.06	0.74
	120	2.35	2.33	2.67	1.77	2.17	3.59	2.61	3.26
*trasnferrin*	0	1.14	0.50	1.74	0.97	1.46	0.73	1.10	0.65
	18	1.72	0.69	1.04	0.66	1.50	0.61	0.99	0.37
	24	1.89	0.98	1.23	0.73	1.76	1.14	1.40	0.47
	36	1.56	0.70	1.03	0.36	1.86	1.16	1.20	0.49
	120	9.89	8.92	10.81	7.48	10.76	11.12	21.92	10.50
*hepcidin*	0	0.92	1.40	2.82	2.52	1.40	1.21	1.37	1.73
	18	1.02	1.12	2.12	2.89	0.82	0.31	0.55	0.23
	24	1.50	2.59	1.70	1.71	2.35	2.40	0.96	0.43
	36	1.31	1.22	1.43	0.88	4.67	11.64	0.67	0.54
	120	1320.33	1673.60	917.26	699.12	659.76	355.95	1564.23	1757.90
*il-1β*	0	1.77	3.08	1.36	1.05	1.50	1.69	1.51	1.80
	18	2.65	4.09	1.84	2.88	1.14	0.83	0.73	0.49
	24	1.12	0.81	0.65	0.41	1.77	1.67	1.03	0.29
	36	1.40	1.49	1.02	0.48	1.22	0.98	0.73	0.49
	120	43.22	39.27	20.67	16.47	22.23	17.56	64.18	52.47
*il-8*	0	0.76	0.77	0.61	0.20	1.55	1.67	0.69	0.38
	18	1.04	1.04	0.96	1.13	0.67	0.36	0.45	0.17
	24	0.73	0.63	0.69	0.65	1.58	1.48	0.81	0.54
	36	1.31	1.54	0.58	0.25	0.99	0.68	0.64	0.56
	120	21.33	14.37	11.18	8.94	12.34	12.47	28.56	21.35
*ccl4*	0	0.98	0.49	1.11	0.31	1.42	1.00	0.97	0.32
	18	1.67	1.71	1.64	2.11	1.25	0.71	1.21	0.66
	24	1.52	0.89	0.95	0.66	1.73	1.01	1.08	0.33
	36	1.15	0.53	1.19	0.28	1.63	0.79	1.11	0.70
	120	2.56	1.83	3.10	2.17	4.17	3.69	4.17	1.66
*il-10*	0	1.22	0.74	1.17	0.40	1.00	0.25	1.22	0.46
	18	1.21	0.36	1.24	0.44	1.09	0.58	1.02	0.22
	24	1.44	0.45	1.26	0.76	1.04	0.26	1.13	0.25
	36	1.10	0.46	1.06	0.43	1.46	0.90	0.89	0.29
	120	7.42	7.60	3.85	3.13	3.26	2.10	5.58	5.39
*hsp70*	0	1.14	0.08	0.99	0.14	0.99	0.15	0.97	0.13
	18	0.85	0.12	0.89	0.23	0.93	0.16	0.97	0.17
	24	1.02	0.34	0.78	0.15	0.90	0.12	0.84	0.10
	36	0.97	0.25	0.87	0.09	0.94	0.70	0.69	0.09
	120	0.95	0.20	1.07	0.13	1.36	1.06	1.40	0.94

**Table A4:** Summary of statistical differences (*p* values) from Tuckey’s multiple comparison test for the relative expression of different genes evaluated in sea bass larvae treated with DnaK and challenged with *V. anguillarum* at different time points (0, 18, 24, 36, and 120 h). Significant differences (*p* < 0.05) are highlighted in bold.

Genes	Group Comparison	Hours Post Challenge				
		0	18	24	36	120
*pentraxin*	NB-MI vs. NB-Ch	0.9483	0.95	0.7373	0.984	0.9998
	NB-MI vs. YS0-Ch	0.5548	>0.9999	0.7564	0.968	>0.9999
	NB-MI vs. YS1-Ch	0.9665	0.9958	0.401	0.9748	0.2522
	NB-MI vs. YS2-Ch	0.9984	>0.9999	0.9666	0.8925	0.5979
	NB-Ch vs. YS0-Ch	0.9384	0.9382	>0.9999	0.771	>0.9999
	NB-Ch vs. YS1-Ch	>0.9999	0.997	0.9926	>0.9999	0.2062
	NB-Ch vs. YS2-Ch	0.8134	0.93	0.3555	0.6113	0.5171
	YS0-Ch vs. YS1-Ch	0.8761	0.9923	0.9902	0.7058	0.3046
	YS0-Ch vs. YS2-Ch	0.2969	>0.9999	0.3744	0.9987	0.6318
	YS1-Ch vs. YS2-Ch	0.8463	0.991	0.1174	0.529	0.9621
*lysozyme*	NB-MI vs. NB-Ch	0.9816	0.9988	>0.9999	0.9996	**0.0249**
	NB-MI vs. YS0-Ch	0.9057	0.8752	0.9996	0.9942	**0.0157**
	NB-MI vs. YS1-Ch	0.7512	0.9204	0.9469	0.9979	0.0695
	NB-MI vs. YS2-Ch	0.3662	0.9833	0.997	0.9998	**0.0063**
	NB-Ch vs. YS0-Ch	0.998	0.9614	0.9977	0.9997	0.9895
	NB-Ch vs. YS1-Ch	0.9674	0.8196	0.9714	0.9866	0.9987
	NB-Ch vs. YS2-Ch	0.693	0.999	0.9994	>0.9999	0.9932
	YS0-Ch vs. YS1-Ch	0.9965	0.4253	0.8727	0.947	0.9537
	YS0-Ch vs. YS2-Ch	0.8291	0.9933	0.9803	0.9993	>0.9999
	YS1-Ch vs. YS2-Ch	0.9549	0.6697	0.9933	0.9874	0.9589
*transferrin*	NB-MI vs. NB-Ch	>0.99	>0.99	>0.99	>0.99	**<0.001**
	NB-MI vs. YS0-Ch	>0.99	>0.99	>0.99	0.99	**<0.001**
	NB-MI vs. YS1-Ch	>0.99	>0.99	>0.99	>0.99	**<0.001**
	NB-MI vs. YS2-Ch	>0.99	>0.99	>0.99	>0.99	**<0.001**
	NB-Ch vs. YS0-Ch	>0.99	>0.99	>0.99	>0.99	0.99
	NB-Ch vs. YS1-Ch	>0.99	>0.99	>0.99	>0.99	0.99
	NB-Ch vs. YS2-Ch	>0.99	>0.99	>0.99	>0.99	**<0.001**
	YS0-Ch vs. YS1-Ch	>0.99	>0.99	>0.99	0.99	>0.99
	YS0-Ch vs. YS2-Ch	>0.99	>0.99	>0.99	>0.99	**<0.001**
	YS1-Ch vs. YS2-Ch	>0.99	>0.99	>0.99	>0.99	**<0.001**
*hepcidin*	NB-MI vs. NB-Ch	>0.9999	>0.9999	>0.9999	>0.9999	**<0.0001**
	NB-MI vs. YS0-Ch	>0.9999	>0.9999	>0.9999	>0.9999	**0.0039**
	NB-MI vs. YS1-Ch	>0.9999	>0.9999	>0.9999	>0.9999	**0.0368**
	NB-MI vs. YS2-Ch	>0.9999	>0.9999	>0.9999	>0.9999	**<0.0001**
	NB-Ch vs. YS0-Ch	>0.9999	>0.9999	>0.9999	>0.9999	0.5799
	NB-Ch vs. YS1-Ch	>0.9999	>0.9999	>0.9999	>0.9999	0.0659
	NB-Ch vs. YS2-Ch	>0.9999	>0.9999	>0.9999	>0.9999	0.8506
	YS0-Ch vs. YS1-Ch	>0.9999	>0.9999	>0.9999	>0.9999	0.8799
	YS0-Ch vs. YS2-Ch	>0.9999	>0.9999	>0.9999	>0.9999	0.1113
	YS1-Ch vs. YS2-Ch	>0.9999	>0.9999	>0.9999	>0.9999	**0.0022**
*il-1β*	NB-MI vs. NB-Ch	>0.99	>0.99	>0.99	>0.99	**<0.001**
	NB-MI vs. YS0-Ch	>0.99	>0.99	>0.99	>0.99	0.05
	NB-MI vs. YS1-Ch	>0.99	>0.99	>0.99	>0.99	**0.02**
	NB-MI vs. YS2-Ch	>0.99	>0.99	>0.99	>0.99	**<0.001**
	NB-Ch vs. YS0-Ch	>0.99	>0.99	>0.99	>0.99	**0.01**
	NB-Ch vs. YS1-Ch	>0.99	>0.99	>0.99	>0.99	**0.02**
	NB-Ch vs. YS2-Ch	>0.99	>0.99	>0.99	>0.99	**0.02**
	YS0-Ch vs. YS1-Ch	>0.99	>0.99	>0.99	>0.99	>0.99
	YS0-Ch vs. YS2-Ch	>0.99	>0.99	>0.99	>0.99	**<0.001**
	YS1-Ch vs. YS2-Ch	>0.99	>0.99	>0.99	>0.99	**<0.001**
*il-8*	NB-MI vs. NB-Ch	>0.99	>0.99	>0.99	>0.99	**<0.001**
	NB-MI vs. YS0-Ch	>0.99	>0.99	>0.99	>0.99	**0.01**
	NB-MI vs. YS1-Ch	>0.99	>0.99	>0.99	>0.99	**<0.001**
	NB-MI vs. YS2-Ch	>0.99	>0.99	>0.99	>0.99	**<0.001**
	NB-Ch vs. YS0-Ch	>0.99	>0.99	>0.99	>0.99	**0.01**
	NB-Ch vs. YS1-Ch	>0.99	>0.99	>0.99	>0.99	**0.02**
	NB-Ch vs. YS2-Ch	>0.99	>0.99	>0.99	>0.99	0.08
	YS0-Ch vs. YS1-Ch	>0.99	>0.99	>0.99	>0.99	>0.99
	YS0-Ch vs. YS2-Ch	>0.99	>0.99	>0.99	>0.99	**<0.001**
	YS1-Ch vs. YS2-Ch	>0.99	>0.99	>0.99	>0.99	**<0.001**
*ccl4*	NB-MI vs. NB-Ch	>0.9999	0.6909	0.7139	0.9999	**0.031**
	NB-MI vs. YS0-Ch	0.9992	0.7504	0.9997	0.9994	**0.0054**
	NB-MI vs. YS1-Ch	0.9346	0.9821	0.4441	0.8471	**<0.0001**
	NB-MI vs. YS2-Ch	>0.9999	0.989	0.9917	>0.9999	**<0.0001**
	NB-Ch vs. YS0-Ch	0.9992	>0.9999	0.8898	>0.9999	0.9099
	NB-Ch vs. YS1-Ch	0.9346	0.9434	0.9956	0.9198	**0.0481**
	NB-Ch vs. YS2-Ch	>0.9999	0.9243	0.9315	>0.9999	**0.0369**
	YS0-Ch vs. YS1-Ch	0.979	0.9625	0.7075	0.9258	0.4689
	YS0-Ch vs. YS2-Ch	0.999	0.9481	0.9996	0.9999	0.437
	YS1-Ch vs. YS2-Ch	0.9248	>0.9999	0.7538	0.8713	>0.9999
*il-10*	NB-MI vs. NB-Ch	0.9995	0.9969	0.9982	0.9997	**<0.0001**
	NB-MI vs. YS0-Ch	0.9998	0.9964	>0.9999	0.9991	0.1938
	NB-MI vs. YS1-Ch	>0.9999	0.9995	>0.9999	0.9998	0.4035
	NB-MI vs. YS2-Ch	0.9995	>0.9999	>0.9999	0.993	**0.0003**
	NB-Ch vs. YS0-Ch	>0.9999	>0.9999	0.9998	>0.9999	**0.009**
	NB-Ch vs. YS1-Ch	0.9994	>0.9999	0.9929	0.996	**0.0004**
	NB-Ch vs. YS2-Ch	>0.9999	0.9996	0.9974	0.9995	0.3103
	YS0-Ch vs. YS1-Ch	0.9998	0.9999	0.9995	0.9923	0.9832
	YS0-Ch vs. YS2-Ch	>0.9999	0.9995	>0.9999	0.9997	0.4897
	YS1-Ch vs. YS2-Ch	0.9994	>0.9999	>0.9999	0.9715	0.1339
*hsp70*	NB-MI vs. NB-Ch	>0.9999	0.9556	0.9703	0.9901	0.9902
	NB-MI vs. YS0-Ch	0.9112	0.9921	0.2385	>0.9999	0.814
	NB-MI vs. YS1-Ch	0.9081	0.9996	0.6239	0.9972	**0.0352**
	NB-MI vs. YS2-Ch	0.8633	>0.9999	0.3933	0.7379	**0.0124**
	NB-Ch vs. YS0-Ch	0.9112	0.9993	0.651	0.9745	0.9652
	NB-Ch vs. YS1-Ch	0.9081	0.9889	0.9568	0.9999	0.1275
	NB-Ch vs. YS2-Ch	0.8633	0.9543	0.8359	0.4585	0.0582
	YS0-Ch vs. YS1-Ch	>0.9999	0.9993	0.9467	0.9894	0.5684
	YS0-Ch vs. YS2-Ch	0.9999	0.9912	0.9947	0.7928	0.4038
	YS1-Ch vs. YS2-Ch	>0.9999	0.9994	0.9962	0.4974	0.9992
